# Modulating the extracellular matrix to treat wound healing defects in Ehlers-Danlos syndrome

**DOI:** 10.1016/j.isci.2024.110676

**Published:** 2024-08-06

**Authors:** Kindra M. Kelly-Scumpia, Maani M. Archang, Prabhat K. Purbey, Tomohiro Yokota, Rimao Wu, Jackie McCourt, Shen Li, Rachelle H. Crosbie, Philip O. Scumpia, Arjun Deb

**Affiliations:** 1 Division of Cardiology, Department of Medicine, David Geffen School of Medicine, University of California, Los Angeles, Los Angeles, CA 90095, USA; 2 UCLA Cardiovascular Theme, David Geffen School of Medicine, University of California, Los Angeles, Los Angeles, CA 90095, USA; 3 Department of Molecular, Cell and Developmental Biology, College of Letters and Sciences, University of California, Los Angeles, Los Angeles, CA 90095, USA; 4 Eli & Edythe Broad Center of Regenerative Medicine and Stem Cell Research, University of California, Los Angeles, Los Angeles, CA 90095, USA; 5 Molecular Biology Institute, University of California, Los Angeles, Los Angeles, CA 90095, USA; 6 California Nanosystems Institute, University of California, Los Angeles, Los Angeles, CA 90095, USA; 7 Department of Integrative Biology and Physiology, University of California, Los Angeles, Los Angeles, CA 90095, USA; 8 Bioengineering Department, University of California, Los Angeles, Los Angeles, CA, USA; 9 Division of Dermatology, Department of Medicine, David Geffen School of Medicine, University of California, Los Angeles, Los Angeles, CA 90095, USA; 10 Medical Scientist Training Program, David Geffen School of Medicine, University of California, Los Angeles, Los Angeles, CA 90095, USA; 11 Department of Dermatology, VA Greater Los Angeles Healthcare System-West Los Angeles, Los Angeles, CA 90073, USA

**Keywords:** medicine, cell biology

## Abstract

Classic Ehlers-Danlos syndrome (cEDS) is a genetic disorder of the connective tissue that is characterized by mutations in genes coding type V collagen. Wound healing defects are characteristic of cEDS and no therapeutic strategies exist. Herein we describe a murine model of cEDS that phenocopies wound healing defects seen in humans. Our model features mice with conditional loss of *Col5a1* in *Col1a2*^+^ fibroblasts (Col5a1CKO). This model shows that an abnormal extracellular matrix (ECM) characterized by fibrillar disarray, altered mechanical properties, and decreased collagen deposition contribute to the wound healing defect. The cEDS animals exhibit decreased expression of epidermal genes and increased inflammation. Finally, we demonstrate that inhibiting mechanosensitive integrin signaling or by injecting wild-type (WT) fibroblasts into cEDS animals enhances epidermal gene expression, decreases inflammation, and augments wound closure. These findings suggest that cell delivery and/or blocking integrin signaling are potentially therapeutic strategies to rescue wound healing defects in cEDS.

## Highlights

• Deletion of *Col5a1* in fibroblasts impairs wound healing by disrupting ECM formation
• Col5a1^−/−^ wounds show elevated inflammation and enhanced integrin expression
• High integrin expression can be targeted by cilengitide to improve wound healing
• Wound healing defects can also be rescued by cell delivery of wild-type fibroblasts

## Graphical abstract

**Figure:**
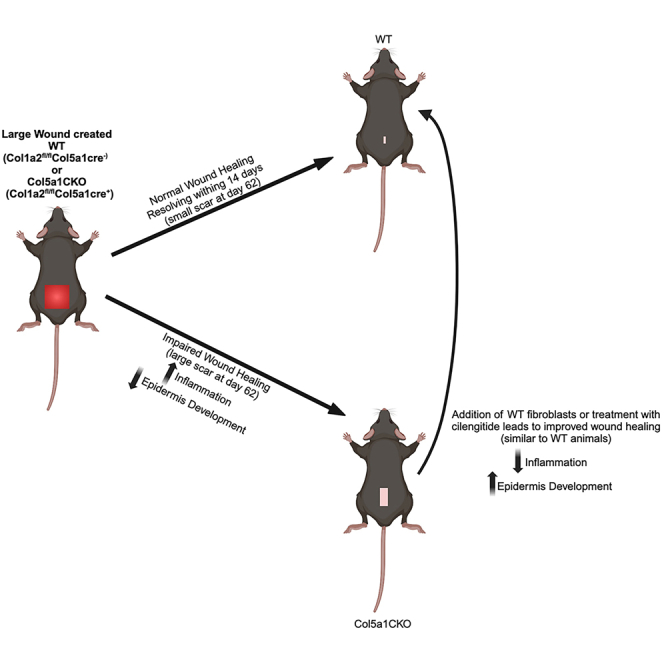

## Introduction

Ehlers-Danlos syndrome (EDS) comprises a group of genetic disorders of the connective tissue and musculoskeletal system affecting 1/5,000–1/25,000 individuals.^1^ Classical EDS (cEDS) is one of the most common (1/10,000–1/20,000) and well-described forms of EDS caused by mutations in *Col5a1* or *Col5a2* genes, and most individuals have decreased type V collagen in tissues.^1^^,^^2^ Type V collagen is classified as a fibril-forming collagen and functions to arrange type I and type III collagen fibrils in a lattice-like framework in the extracellular matrix (ECM) and is required for the proper architecture of the ECM in the skin and other tissue.^3^^,^^4^^,^^5^ For instance, in the complete absence of ColV, mesenchyme is unable to form with early death of the embryo. ColV is also known to play an important role in arranging the fibrillar pattern of the ECM after tissue injury, and deficiency of ColV after corneal wounding results in loss of optical transparency of the cornea. The functional role of ColV in maintaining the normal architecture of the matrix and other tissue contributes to the characteristic phenotype observed in individuals with cEDS who are typically haploinsufficient. Phenotypically, cEDS is characterized by skin hyperextensibility, joint hypermobility, fragile skin, easy bruising, and delayed wound healing with formation of atrophic scars.^6^ Individuals with cEDS demonstrate a profound defect in wound healing, and defects in wound healing are so common that impaired wound healing is considered to be a major criterion for the diagnosis of cEDS.^6^ The lack of a normal ECM architecture after skin wounding in cEDS results in failure of epithelial regeneration in individuals with cEDS and this results in the formation of broad scars. The scars (“cigarette paper-like”) lack the normal tensile strength of scars in normal tissue and break down predisposing the affected individual to infections, bleeding, and further scarring.^7^^,^^8^ Despite the immense importance of wound healing defects in individuals with cEDS, little is known about the underlying mechanisms of defective wound healing in cEDS. Skin fibroblasts after skin wounding express a variety of collagens including ColV, and whether the secretion of ECM proteins deficient in ColV after skin wounding contributes to wound healing defects or whether the abnormal matrix present prior to wounding in ColV-deficient animals predominantly contributes to wound healing defects is not clear. Currently, there are no therapeutic targets or strategies to treat the wound healing defects in cEDS. Determining the role of fibroblasts and their secretion of ColV-deficient matrix in wound healing defects in cEDS could help in identifying new targets that could be administered after wounding to attenuate wound healing defects.^9^

Animal models of cEDS have been described that use a haploinsufficient Col5a1^+/−^ mouse that displays delayed wound healing compared to Col5a1^+/+^ mice in a small, unsplinted wounding model. In this model, *Col5a1* abnormalities of the matrix are present from birth making it difficult to ascertain the active and direct role of *Col5a1* in wound healing.^10^ While these cultured haploinsufficient fibroblasts displayed decreased proliferation and migration *in vitro*,^10^ it would not be possible to tease out the role of acute production of type V collagen versus preexisting matrix dysfunction using this model.

To more directly address the question of the role of ColV secretion after skin wounding in contributing to defective wound healing, we created a murine model of cEDS, where the role of *Col5a1* in wound healing can be more directly ascertained. In this model, we conditionally deleted Col5a1 in Col1a2-expressing fibroblasts at the time of skin wounding to directly ascertain the role of ColV secretion at the time of wounding, in contributing to cEDS wound healing defects.^11^ This was done by crossing Col1a2CreERT animals with animals that had both Col5a1 alleles floxed. As the Cre-mediated deletion of Col5a1 is regulated by a tamoxifen inducible cassette, this enables us to precisely determine the role of ColV at the time of skin wounding.

We show that acute loss of *Col5a1* in fibroblasts at the time of skin wounding is sufficient to phenocopy wound healing defects in individuals with cEDS with delayed re-epithelialization, poor ECM organization, and enhanced and prolonged inflammation. These defects are associated with differential expression of integrins in Col5a1CKO wounds that also have been observed in individuals with cEDS. Using this model, we identify abnormalities of wound mechanics, inflammation, and dysregulated gene expression that underlies defects in cEDS. We demonstrate that rescue of ECM at the time of wounding with fibroblast transplantation or small molecules is sufficient to rescue the phenotype of defective wound healing in cEDS. Our observations have potential ramifications for designing therapeutic strategies for wound healing defects in cEDS.

## Results

### A murine model of cEDS that phenocopies wound healing defects in human cEDS

**Figure 1:**
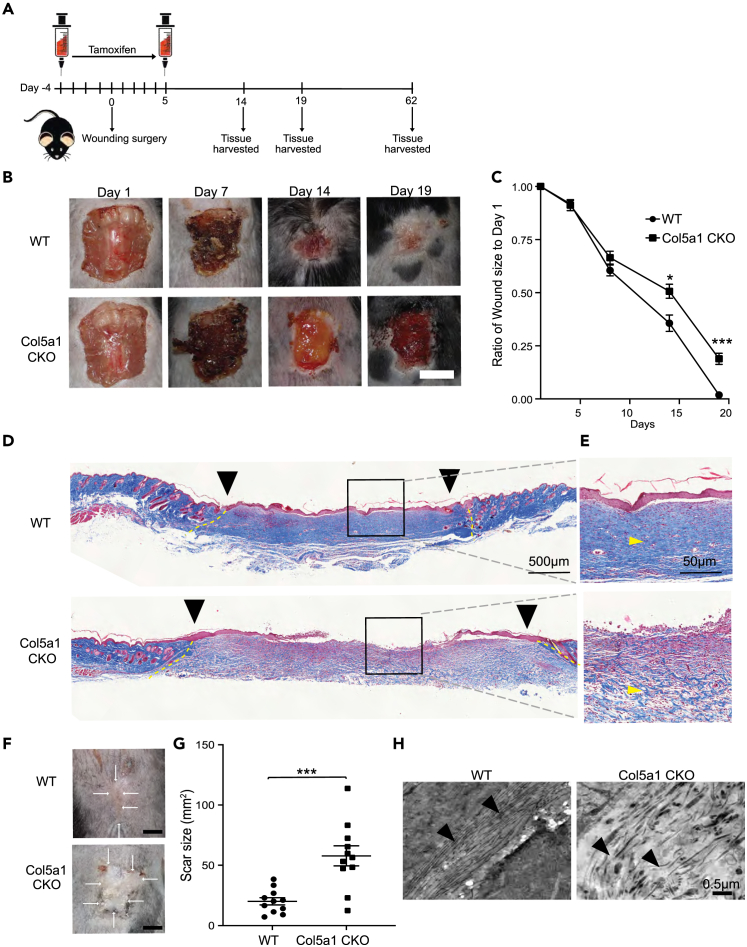
Impaired wound healing in Col5a1CKO mice (A) Experimental design of skin wound injury model demonstrating timing of tamoxifen and follow up. (B) Representative pictures of mouse wounds in WT (Cola2Cre^−^:Col5a1^fl/fl^) and Col5a1CKO animals over time (scale bar represents 50 mm). (C) Ratio of wound size normalized to wound size at time of injury (data are represented as mean ± SEM; WT *n* = 11 [6M, 5F]; Col5a1CKO *n* = 11 [5M, 6F]; two-way ANOVA Sidak’s multiple comparisons test ∗*p* < 0.05; ∗∗∗*p* = 0.003). (D) Representative image of Masson’s trichrome staining of WT and Col5a1CKO mouse at day 19 (5×, at least *n* = 3 per group). Yellow dashed lines mark wound edges. (E) Gray dashes show inlet of higher magnification (20×). (F) Representative scar picture of WT and Col5a1CKO mice at day 62. (G) Quantitation of scar size (WT *n* = 11 [6M, 5F]; Col5a1CKO *n* = 11 [5M, 6F]; line represents mean±SEM; Student’s t test *p* < 0.001). (H) Representative transmission electron microscopy (TEM) of wound showing fibrillar disarray in Col5a1CKO (arrowhead showing disarray; scale bar represents 0.5 μM), at least *n* = 3 per group).

To investigate the biology of wound healing defects in cEDS, we engineered a mouse model of cEDS by conditionally deleting *Col5a1* in skin fibroblasts at the time of skin wounding. For this purpose, we crossed animals harboring an inducible fibroblast Cre driver (*Col1a2*CreERT), with animals that have both *Col5a1* genes floxed.^4^ The *Col1a2*CreERT driver has been shown previously to efficiently label skin fibroblasts and the animal was generated by insertion of a CreERT2 (tamoxifen inducible Cre cassette) downstream of a pro alpha 2 (I) collagen enhancer.^12^^,^^13^ Progeny animals (harboring the Cre transgene as well as homozygous for the floxed alleles; *Col1a2*CreERT: *Col5a1*fl/fl) were then subjected to skin wounding. Skin wounding was achieved by 2.25 cm^2^ full thickness excision biopsies on the dorsum of the animal. The size was chosen as cutaneous wounds in adult mice that are smaller than 1.56 cm^2^ can heal entirely by wound contracture, while the final 0.5 cm^2^ of 2.25 cm^2^ wounds consistently heal with re-epithelialization and fibrosis in the center.^14^ Tamoxifen was administered starting 4 days prior to skin wounding and continued for 5 days after to induce Cre-mediated recombination in skin fibroblasts (Figure 1A). Control animals included Cre^−^:Col5a1^fl/fl^ littermates (referred to as wild-type [WT] henceforth) and were also subjected to identical wounding and tamoxifen administration. *Col5a1* gene expression was confirmed to be decreased in the skin of Col5a1CKO animals by qPCR and RNA sequencing (RNA-seq) (S1). Imaging and tissue harvests were then performed at different time points to determine the repair response to skin wounding (Figure 1A). We observed that compared to control WT animals, whose wounds grew smaller and closed over time, the wounds of Col5a1CKO animals were significantly larger (Figure 1B) and nearly 25% of the initial wound size was still open and unhealed in the Col5a1CKO animals by day 19 after injury (Figure 1C). We next performed histological analysis of the injured region and observed that the wounded bed was completely covered by the epidermis in the WT littermates but there was failure of epidermal reconstitution of the wound bed in the Col5a1CKO animals (Figure 1D). Masson trichrome staining demonstrated a loose organization of the granulation tissue of the injury region in Col5a1CKO animals compared to the control littermates (Figure 1E). At 62 days after tissue injury, the area of scarring was significantly greater in the Col5a1CKO animals compared to the WT littermates (Figures 1F and 1G). We have previously demonstrated that type V collagen is a critical determinant of fibrillar organization of the matrix in the injured region.^11^ To determine whether collagen fibrillar organization was disrupted, we performed transmission electron microscopy of the wounded skin bed and observed parallel arrangement of collagen fibrils in the WT animals but gross fibrillar disorganization in the Col5a1CKO animals with a haphazard arrangement of collagen fibrils running in directions orthogonal to each other (Figure 1H). Taken together, these observations demonstrate the Col5a1CKO animal after skin wounding phenocopies wound healing defects in human cEDS with disrupted wound healing, broad scarring, and fibrillar disorganization of the injury region.

### Defective wound healing in Col5a1CKO animal is associated with decreased expression of epidermal genes and increased inflammation

**Figure 2:**
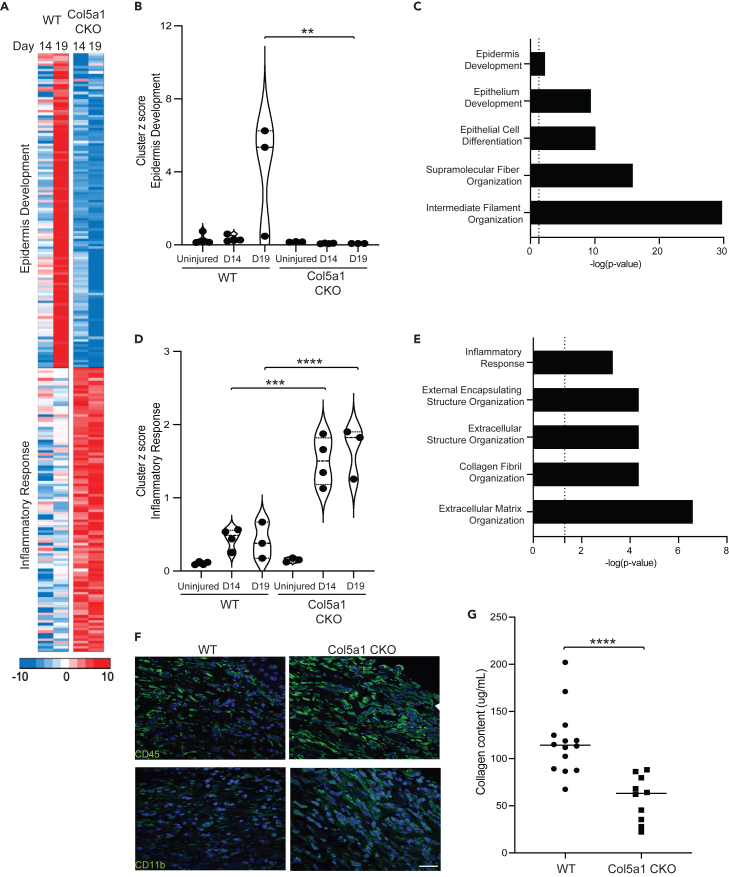
Col5a1CKO mice exhibit altered expression of epidermal and inflammatory genes following skin wounding (A) Heatmap showing significant K mean clusters for genes (*p* < 0.05) differentially expressed (at least 2-fold difference in expression at day 14 and day 19) between wounded WT and Col5a1CKO mice. Gene enrichment analysis allows us to label them epidermis development and inflammatory response. (B) *Z* score analysis of genes in epidermis development (Uninj = unwounded skin; *n* = 2 for day 14 and day 19, WT day 14 *n* = 4 [2M, 2F]; WT day 19 *n* = 3 [1M, 2F]; Col5a1CKO day 14 *n* = 4 [2M, 4F]; Col5a1CKO day 19 *n* = 3 [1M, 2F]; one-way ANOVA was performed with Tukey post hoc analysis; ∗∗*p* < 0.01, ∗∗∗*p* < 0.001, ∗∗∗∗*p* < 0.0001). (C) Results of gene enrichment analysis for cluster by −log *p* value. (D) *Z* score analysis of genes in inflammatory response (Uninjured = unwounded skin; *n* = 3 for day 14 and day 19, WT and Col5a1CKO *n* = 4 at day 14 and *n* = 3 at day 19 after wounding; one-way ANOVA was performed with Tukey post hoc analysis; ∗∗*p* < 0.01, ∗∗∗*p* < 0.001, ∗∗∗∗*p* < 0.0001). (E) Results of gene enrichment analysis for cluster by *p* value. (F) Representative (*n* = 3–5 per group; scale bar represents 50 mm) immunofluorescent images of CD45 and CD11b cells (arrows) in wounds of WT vs. Col5a1CKO animals demonstrating an increase in cell infiltrates in Col5a1CKO mice. (G) Total collagen by biochemical determination in wounds at day 14 (WT *n* = 14 [6M, 8F]; Col5a1CKO *n* = 10 [5M, 5F]; line indicates mean; unpaired test *p* < 0.0001).

To obtain insight into underlying mechanisms causing defective wound healing, we performed gene expression analysis of the injured tissue at 14 days and 19 days following skin wounding. A K means cluster was performed on differentially expressed genes (*p* < 0.05) that demonstrated at least a 2-fold difference in gene expression between WT and Col5a1CKO animals using Cluster 3.0. We observed two different clusters of genes, epidermal and inflammatory genes, to be significantly up- and downregulated between the wounds of WT and the Col5a1CKO animals (Figure 2A). Gene ontogeny analysis demonstrated that genes related to epidermis development, epithelial differentiation, and filament organization were significantly upregulated in injured tissue of WT animals compared to uninjured animals, but epidermal genes did not demonstrate any significant upregulation in the Col5a1CKO animal after injury (Figures 2B and 2C). In contrast, gene ontogeny demonstrated that expression of inflammatory genes was significantly upregulated in the injured tissues of Col5a1CKO animals compared to WT littermates (Figures 2D and 2E). ECM organization was another pathway identified to be upregulated in Col5a1CKO animals. These genes included matrix metalloproteinases known to degrade the ECM (Figure 2E). Immunostaining confirmed increased accumulation of CD45^+^ and CD11b^+^ inflammatory cells in the tissues of Col5a1CKO animals at day 19 after tissue injury (Figures 2F and S3). As histology of the injured region had demonstrated loose organization of granulation tissue in the Col5a1CKO, we next measured collagen content using biochemical assays and observed that total collagen content was significantly decreased in the Col5a1CKO animals compared to the WT littermates (Figure 2G).

### Modulation of integrin signaling or injection of WT fibroblasts into wound bed of Col5a1CKO injured tissues to attenuate wound healing defects

**Figure 3:**
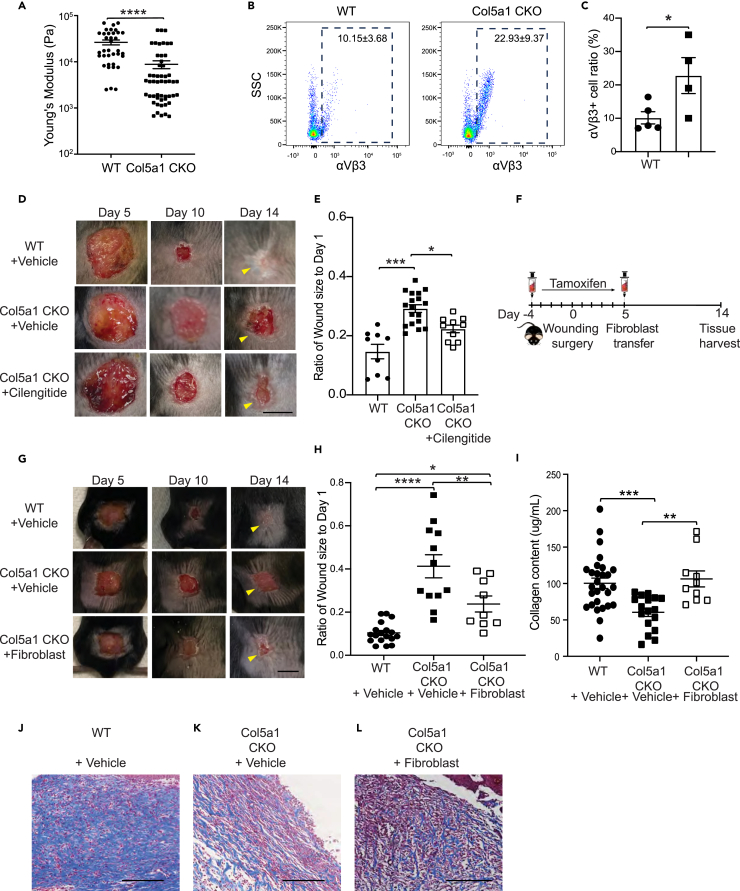
Cilengitide or injection with WT fibroblasts attenuates wound healing defects in Col5a1CKO mice (A) Young’s modulus measurements from injured regions (36 measurements for WT and 52 for Col5a1CKO from at least 3 different mice per group; line indicates mean±SEM Mann-Whitney test *p* < 0.0001). (B) Representative flow dot plots of αvβ3 by in day 14 wounds of WT and Col5a1CKO animals. (C) Quantification of % of αvβ3 CD45^−^ cells in skin of WT and Col5a1CKO animals via flow cytometry (WT *n* = 4 [2M, 3F]; Col5a1CKO *n* = 4 [2M, 2F]; error bars represent SEM; unpaired t test). (D) Representative images of mouse wounds in WT and Col5a1CKO animals treated with vehicle or cilengitide (scale bar represents 50 mm). (E) Wound size (normalized to initial wound size) demonstrating augmented healing in cilengitide treated Col5a1CKO animals at day 10 (WT *n* = 9 [4M, 5F]; Col5a1CKO *n* = 18 [8M, 10F]; Col5a1CKO + cilengitide *n* = 10 [4M, 6F]; error bars represent SEM; one-way ANOVA with Holms-Sidak’s multiple comparisons test ∗∗*p* < 0.01; ∗∗∗*p* < 0.00001). (F) Experimental design showing timing for treatment of animals with tamoxifen and the injection of WT fibroblasts into skin wounds. (G) Representative images of mouse wounds over time (scale bar represents 50 mm) for WT, Col5a1CKO + vehicle, or Col5a1CKO + fibroblast-treated animals. (H) Wound size on day 14 demonstrating augmented healing in Col5a1CKO mice injected with fibroblasts (WT *n* = 18; Col5a1CKO *n* = 11; Col5a1CKO + cilengitide *n* = 10; line represents mean±SEM one-way ANOVA with Holms-Sidak’s multiple comparisons test ∗*p* = 0.0256; ∗∗*p* = 0.006; ∗∗∗∗*p* < 0.0001). (I) Total collagen content in wounds at day 14; line represents mean±SEM (one-way ANOVA with Holms-Sidak’s multiple comparisons test ∗∗∗*p* = 0.0009, ∗∗*p* = 0.0022; *n* = 28 WT + vehicle; *n* = 17 Col5a1 CKO+vehicle; *n* = 10 Col5a1CKO + fibroblast). (J–L) Masson trichrome staining of wounds. (J) WT + vehicle, (K) Col5a1CKO + vehicle, and (L) Col5a1CKO + fibroblasts on day 14 (*n* = 3 per group) demonstrating fibrotic collagen in healed wounds of WT mice and granulation tissue in unhealed wounds of both Col5a1CKO groups. Col5a1CKO animals that received fibroblasts demonstrated thicker dermal granulation tissue with more collagen and higher cellularity than Col5a1CKO mice that did not receive fibroblasts (scale bar represents 100 mm).

We have previously demonstrated that injured cardiac tissue deficient in type V collagen have abnormal mechanical properties.^11^ The matrix of the injured skin tissue of the Col5a1CKO demonstrated fibrillar disarray, and we performed atomic force microscopy (AFM) of the tissue bed at day 14 and observed a significantly lower Young’s modulus or stiffness (Figure 3A). This is consistent with the abnormal architecture of the matrix as well as decreased collagen deposition observed in the wounds of Col5a1CKO animals. We had also observed that in the heart, abnormal matrix formation was in part determined by mechanosensitive integrin dependent signaling.^11^ Blockade of alpha v beta 3 (***α***v***β***3) with the small molecule cilengitide led to rescue of matrix abnormalities and partial rescue of the defective wound healing observed in the type V collagen-deficient heart tissue.^11^ Individuals with EDS are known to harbor increased expression of ***α***v***β***3 in skin myofibroblasts.^15^ We performed flow cytometry of injured skin of WT and Col5a1CKO animals at 7 days after wounding to determine expression of **α**v**β**3 and observed significant increased expression of αvβ3 in cells isolated from the injured skin of Col5a1CKO animals compared to WT controls (Figures 3B and 3C). As blockade of ***α***v**β**3 signaling with cilengitide had augmented repair in the heart deficient in type V collagen,^11^ we next determined whether administration of cilengitide would rescue wound healing defects of the skin in the Col5a1CKO animal. For this purpose, we subjected the Col5a1CKO animal to skin wounding and injected cilengitide (20 mg/kg intraperitoneal [i.p.]) from days −1 to 13 after skin wounding. Animals were imaged serially over 14 days (Figure 3D) and we observed that cilengitide significantly enhanced wound closure in Col5a1CKO animals compared to injured Col5a1CKO animals injected with vehicle at day 14 (Figure 3E). These observations are consistent with our earlier published observations demonstrating the modulation of ***α***v***β***3 mechanosensitive signaling enhances wound healing defects in Col5a1CKO animals.

As the ECM depleted of type V collagen is abnormal with fibrillar disarray, we hypothesized that injection of WT fibroblasts into wounds of Col5a1CKO animals should partially rescue wound healing defects. For this purpose, we isolated fibroblasts from the skin of WT animals and injected 5 × 10^6^ fibroblasts intradermally into the wound bed of Col5a1CKO animals at 3 days after skin wounding and harvested the animals at day 14 to determine the effects of injecting WT fibroblasts on wound repair (Figure 3F). We observed that Col5a1CKO animals exhibited a marked defect in wound healing with unhealed wounds, but by day 14, the Col5a1CKO animals that received the WT fibroblasts exhibited significant attenuation of wound healing defects (Figures 3G and 3H). The Col5a1CKO animals that received the fibroblasts had a 50% significant improvement in the wound size by day 14 compared to vehicle-injected Col5a1CKO animals (Figure 3H) We also compared Col5a1CKO fibroblasts transferred into Col5a1CKO animals and did not observe any improvement in wound healing in Col5a1CKO wound animals injected with Col5a1CKO fibroblasts (S2). Biochemical measurement of collagen content demonstrated a complete rescue of the deficits in collagen deposition in the Col5a1CKO animals that received the fibroblasts and the amount of collagen deposited was not significantly different than the WT littermates (Figure 3I). Masson trichrome staining of the injured tissue bed demonstrated fibrotic collagen in healed wounds of WT + vehicle mice (Figure 3J) and granulation tissue in unhealed wounds of Col5a1CKO animals with and without the addition of WT fibroblasts (Figures 3K and 3L). Col5a1CKO mice that received fibroblasts displayed thicker dermal granulation tissue with more collagen and higher cellularity than Col5a1CKO mice that did not receive fibroblasts (Figure 3L).

### Injection of fibroblasts enhances epidermal gene expression and attenuates inflammatory gene expression in injured tissue of Col5a1CKO animals

**Figure 4:**
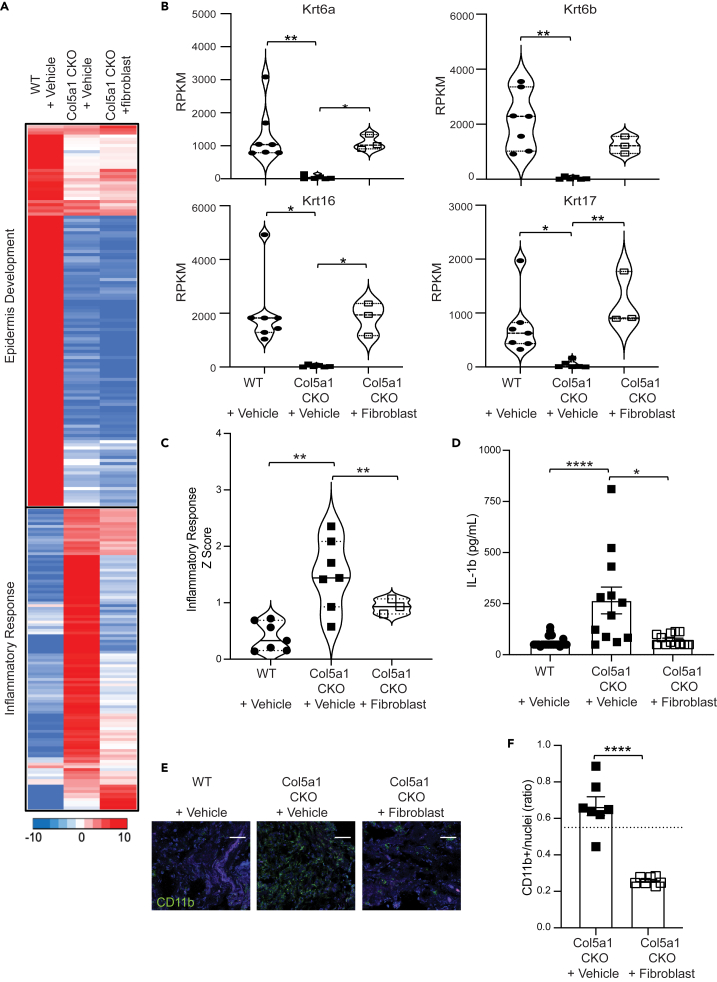
RNA-seq of Col5a1CKO mice receiving WT fibroblasts display increased keratin gene expression and significantly reduced inflammatory response (A) Heatmap showing significant K mean clusters for genes differentially expressed (*p* < 0.05, at least 2-fold difference in expression) in wounds of WT and Col5a1CKO mice at day 14 compared with Col5a1CKO animals treated with WT fibroblasts. (B) Bulk RNA sequencing (RPKM) expression levels for keratin 6a, 6b, 16, and 17. Gene expressions of these genes are significantly improved in mice transplanted with WT fibroblasts. (C) *Z* score showing gene enrichment analysis for inflammatory response cluster by *p* value (WT + vehicle *n* = 7 [4M, 3F]; Col5a1CKO + vehicle *n* = 7 [3M, 4F]; Col5a1CKO + fibroblast *n* = 3 [1M, 2F]). (D) IL-1β cytokine level (day 14) in WT or Col5a1CKO mice treated with vehicle or fibroblasts (Kruskal-Wallis with Dunn’s multiple comparison test; WT + vehicle *n* = 25 [11M, 14F], Col5a1CKO + vehicle *n* = 12 [5M, 7F], Col5a1CKO + fibroblast *n* = 12 [6M, 6F]; Kruskal-Wallis with Dunn’s multiple comparison test ∗*p* < 0.05, ∗∗∗∗*p* < 0.0001). (E) Representative immunofluorescent staining for CD11b (arrows) in day 14 wounds of WT or Col5a1CKO animals treated with vehicle or fibroblasts (*n* = 3 per group; scale bar represents 50 mm). (F) Quantification of CD11b cells in sections, at least 100 cells counted from 3 independent animals (Col5a1CKO + vehicle *n* = 7, Col5a1CKO + fibroblast *n* = 8; error bars represent SEM; unpaired t test *p* < 0.0001).

We next determined whether injection of WT fibroblasts into wounds of Col5a1CKO animals altered the transcriptional response to tissue injury. We induced skin wounding in WT and Col5a1CKO animals and injected Col5a1CKO animals with either vehicle or WT fibroblasts as described earlier (Figure 3F). Tissues were then harvested at 14 days after skin wounding and subjected to bulk RNA-seq. We focused on genes regulating epidermal development and inflammation as we had observed these gene clusters to be differentially expressed in injured tissues of Col5a1CKO versus WT littermates (Figure 4A). For the cluster of epidermal genes, we observed that injection of WT fibroblasts into Col5a1CKO wounds led to significant increase in expression of critical epidermal genes such as Krt6a, Krt6b, Krt16, and Krt17 (Figure 4B). In particular, Krt16/17 have been shown to play a critical role in wound healing of the skin,^16^ and increased expression of these critical keratins in Col5a1CKO animals injected with fibroblasts is consistent with their enhanced wound healing phenotype. The cluster of inflammatory genes that was upregulated in injured tissues of Col5a1CKO animals versus WT animals exhibited significant attenuation of expression in the Col5a1CKO animals that received WT fibroblasts (Figure 4A). The inflammatory transcriptional response measured by a *Z* score was significantly downregulated in Col5a1CKO animals that received fibroblasts compared to WT animals (Figure 4C). We also performed bulk RNA-seq on WT + vehicle, Col5a1CKO + vehicle, and Col5a1CKO + cilengitide at day 14 and saw similar results as the bulk RNA-seq with the addition of WT fibroblasts (Figure S4). Specifically, we observed a decrease in the inflammatory response signature and an increase in the epidermis development signature of Col5a1CKO animals treated with cilengitide compared to Col5a1CKO animals + vehicle. The inflammatory response and epidermis development signatures from Col5a1CKO + cilengitide-treated animals were similar to the signatures seen in WT + vehicle animals. Interleukin 1β (IL-1β) is an inflammatory cytokine that is upregulated in chronic non-healing wounds in humans and in diabetic mice and thought to impair the wound healing response.^17^ We measured IL-1β in injured tissues of Col5a1CKO and WT animals at day 14 after injury and observed significantly increased levels of IL-1 β in injured tissues of Col5a1CKO animals (Figure 4D). However, IL-1β levels in Col5a1CKO animals injected with fibroblasts was significantly decreased compared to Col5a1CKO animals and were not different from those of injured tissues of WT animals (Figure 4D). Immunostaining for inflammatory cells in injured tissues also demonstrated that the fraction of CD11b^+^ cells in the injured region was significantly decreased in the Col5a1CKO animals that received fibroblasts compared to Col5a1CKO animals that received vehicle (Figures 4E, 4F, and S3). Taken together, these observations demonstrate that injection of WT fibroblasts or treatment of Col5a1CKO animals with cilengitide not only leads to augmented wound healing with significantly greater closure of wounds, but favorably alters the transcriptional response to wounding with greater expression of genes associated with epidermis development and attenuation of the inflammatory response.

## Discussion

Defective wound healing is one of the most common and debilitating symptoms in cEDS but no effective treatments yet exist for augmenting wound healing in this condition. We demonstrate that the Col5a1CKO animal remarkably phenocopies human wound healing defects in cEDS. A previously described haploinsufficient model had also demonstrated wound healing defects but in that instance as *Col5a1* had been deleted *in utero*, it was difficult to exclude secondary compensatory changes that could have affected wound healing.

The Col5a1CKO model described by us thus can directly examine the role of type V collagen in wound healing as deletion of *Col5a1* occurs at the time of wounding. We observed that skin wounds demonstrated delayed closure and exhibited decreased mechanical strength and broad scarring. Such a phenotype of defective wound healing is commonly observed in humans with cEDS, thus making the model suitable for investigating the biology of wound healing defects in cEDS. Our data suggest that abnormalities of the matrix with collagen fibrillar disarray, altered mechanical, properties and decreased collagen deposition contribute to wound healing defects in cEDS. Regrowth of the epidermis at the edges of the wound is critical for effective wound closure but the Col5a1CKO animal demonstrated significant downregulation of epidermal growth or development, along with a persistent inflammatory infiltrate. Persistence of inflammation can be commonly observed in individuals with cEDS, often secondary to poor healing or recurrent trauma.^1^ Transcriptomic analysis of wounds of Col5a1CKO animals demonstrated decreased expression of ECM genes and biochemical measurements confirmed decreased collagen deposition. These observations are consistent with recent studies of skin fibroblasts from patients with cEDS that showed decreased expression of canonical genes of fibroblast activation.^9^ Using this murine model that recapitulates wound healing defects in human cEDS, we demonstrate that mechanical properties of the wound are abnormal, with wounds lacking mechanical strength. We show here that the wounds of Col5a1CKO animals are enriched in ***α***v***β***3 expression and those findings are also consistent with human data demonstrating abundant expression of various integrins and specifically **α**v**β**3 in myofibroblasts in skin of humans with cEDS.^15^ In a model of cardiac injury, we have previously demonstrated that cilengitide, which is known to inhibit ***α***v***β***3 signaling, can partially rescue aberrant mechanosensitive integrin signaling and lead to enhanced wound healing in type V collagen-deficient animals.^11^ Consistent with those observations, we demonstrate that the small molecule cilengitide can also partially rescue skin wound healing defects in the Col5a1CKO animal. As the matrix appears to the primary abnormality contributing to defective wound healing in cEDS, we demonstrate that cellular therapy by injection of WT skin fibroblasts into skin wounds of Col5a1CKO animals or the treatment of Col5a1CKO animals with cilengitide can significantly enhance wound healing and reverse adverse changes in gene expression affecting epidermal and inflammatory genes. Wounds of treated Col5a1CKO animals (with either WT fibroblasts or cilengitide) demonstrated rescue of expression of epidermal genes regulating epidermal repair as well as decreased expression of pro-inflammatory genes. The wounds exhibited significantly greater amount of collagen deposition and overall the closure of the wounds was enhanced within 14 days of injury. Both the inflammatory response and epidermis development pathway could be potential targets for improving wound healing. Taken together, these observations demonstrate a murine model of cEDS and provide insight into modulation of integrin-based mechanosensitive signaling as well as cellular injection of fibroblasts that could serve as potential therapeutic strategies for enhancing wound healing in cEDS.

### Limitations of the study

In this study, we demonstrate how loss of type V collagen in wound fibroblasts disrupts the ECM of healing wounds resulting in severe wound healing defects, recapitulating a major feature of the human disease, cEDS. While we show that inhibition of integrin signaling by systemic administration of cilengitide can improve wound healing as a proof of concept, topical administration for wounds would be a preferable treatment modality for cutaneous wounds. These experiments need to be performed in order to translate this treatment modality as a potential therapeutic for patients. In addition, while we show that deletion of *Col5a1* leads to wounds that are more inflamed, whether exaggerated wound inflammation contributes to wound healing defects in this model or cEDS has not been examined in detail and will be evaluated in future experiments.

## Resource availability

### Lead contact

• Further information and requests for resources and reagents should be directed to and will be fulfilled by the Lead Contact, Arjun Deb (adeb@mednet.ucla.edu).

### Materials availability

• This study did not generate new unique reagents.

### Data and code availability

• The bulk RNA sequencing in this paper are deposited in NCBI GEO dataset (GSE264169) and are available as of August 1, 2024.
• This paper does not report original code.
• Any additional information required to reanalyze the data reported in this paper is available from the lead contact upon request.

## Acknowledgments

We thank Dr. Andrew Leask, University of Western Ontario, Canada, and Dr. David Birk, University of South Florida for providing us with the Col1a2CreERT2 and the Col5a1fl/fl mice. We also thank Rakhi Banerjee for histological and technical assistance. This study was supported by grants from the 10.13039/100000002National Institutes of Health, USA (HL149658, HL152176, HL149687, AR075867, and DK132735 to A.D. and AR079470 to P.O.S.) and a grant from the 10.13039/501100012331LEO Foundation, DK (LF-OC-21-000688 to P.S.). M.A. was supported in part by 10.13039/100000002NIH T32-GM008042.

## Author contributions

K.M.K.-S. performed *in vitro* and *in vivo* experiments, histological stains, and other bench experiments and helped with bioinformatic analysis; M.M.A. performed *in vitro* and *in vivo* experiments and histological stains. T.Y. and R.W. performed histological and *in vivo* experiments. J.M. performed atomic force microscopy experiments. P.K.P. analyzed bulk sequencing data and S.L. analyzed flow data and prepared the figures. R.H.C. and P.O.S. supervised experiments and analyzed histology and A.D. conceptualized the project, designed all experiments, interpreted the data, and wrote the manuscript.

## Declaration of interests

The authors declare no conflict of interests.

## STAR★Methods

### Key resources table

**Table:** 

REAGENT or RESOURCE	SOURCE	IDENTIFIER
**Antibodies**		

Mouse anti-integrin aVb3	Abcam	Cat# ab7166; RRID: AB_305742
Recombinant Anti-CD45	Abcam	Cat#ab10558;RRID:AB_442810
Recombinant Anti-CD11b	Abcam	Cat# ab133357; RRID AB_2650514
Goat Anti-Rabbit IgG H&L (Alexa Fluor 594)	Abcam	Cat#ab150080; RRID AB_2650602
Goat Anti-Rabbit IgG H&L (Alexa Fluor 488)	Abcam	Cat#150077; RRID AB_2630356
VECTASHIELD PLUS antifade mounting medium with DAPI	Vector Laboratories	H-2000-10
BD Cytometric Bead Array (CBA) Mouse IL-1β; Flex Set Bead E5	BD Bioscience – Fisher Scientific	Cat #BDB560232
Rabbit IgG, Isotype Control, polyclonal	Abcam	Cat#ab171870; RRID AB_2687657
Rabbit IgG, Isotype Control, monoclonal	Abcam	Cat#ab172730;RRID AB_2687931

**Chemicals, peptides, and recombinant proteins**		

Cilengitide	MCE	#HY-16141
Tamoxifen	Sigma	T5648
Permount Mounting Medium	Fisher Scientific	SP15100
Tissue-Tek OCT	Sakura Finetec	#4583
Liberase DH	Sigma	#5401054001

**Critical commercial assays**		

Sircol Insolulble Collagen Assay	Biocolor	S2000
RNAeasy Mini Kit	Qiagen	74104
Masson Trichrome Stain Kit	Thermo Scientific	87019
RNAseq data from wounded WT and Col5a1 CKO skin	This paper	GSE264169

**Experimental models: Cell lines**		

Mouse primary skin fibroblasts	This paper	N/A

**Experimental models: Organisms/strains**		

Mouse: Col1a2-CreERT:C57BL/6	Zheng et al., 2002	Zheng et al.^12^
Mouse: Col5a1 fl/fl:C57BL/6	Sun et al., 2011	Sun et al.^4^
Mouse: Col1a2-CreERTxCol5a1 fl/fl:C57BL/6	Yokota et al. 2020	Yokota et al.^11^

**Software and algorithms**		

ImageJ	NIH	https://imagej.nih.gov/ij/
FlowJo	FlowJo	https://www.flowjo.com
Prism 10	GraphPad	N/A
R package Seurat	Bioconductor	3.0.2
Enrichr	Chen et al. 2013 Kuleshov et al. 2016	Chen et al.^18^ Kuleshov et al.^19^; https://maayanlab.cloud/Enrichr/
Hisat2	Siren et al. 2014	Sirén et al.^20^
DEseq-2	Bioconductor	NA
Cluster 3.0	Hoon et al. 2004	de Hoon et al.^21^

**Other**		

Nikon Eclipse Ti2 Confocal microscopy	Nikon	https://www.microscope.healthcare.nikon.com/products/inverted-microscopes/eclipse-ti2-series
JEM1200EX transmission electron microscope	JECL	https://www.jeolusa.com/PRODUCTS/Transmission-Electron-Microscopes-TEM
TF20 TEM	FEI	
SHOCONGG-TL AFM Probes	AppNano	SHOCONGG-TL
JPK Nanowizard 4A BioAFM	Bruker, JPK	https://usa.jpk.com/products/atomic-force-microscopy/nanowizardultra-speed-2
TissueLyser LT	Qiagen	Cat. No/Id:85600
LSRFortessa X-20 SORP	BD Bioscience	N/A
Synergy H1 microplate reader	BioTek	N/A

### Experimental model and study participant details

#### Animal care and use

All animal studies were approved by the Animal Research Committee, University of California, Los Angeles. All animals were maintained at the UCLA vivarium according to the policies instituted by the American Association for Accreditation of Laboratory Animal Care. Male and female animals aged between 8 and 12 weeks were used in the study. All animals were healthy, immune-free, and drug or test naive and were not involved in other experimental procedures. Littermates were used as controls for all experiments. For generation of Col5a1CKO mice, Col1a2CreERTmice were crossed with the Col5a1 floxed^12^; B6 background) mice^4^ and progeny mice were administered tamoxifen (1 mg IP daily) for 5 days prior to wounding and continued for 5 days following injury.

#### Murine models of wounding (WIHN model)

We performed a large wound excision model as described.^22^ Mice are anesthetized with isoflurane and 100% oxygen. Ensure the deep pedal reflexes of the mouse are suppressed apply lubricating ointment to the eyes of the animal and place in the prone position. Each mouse was then administered a dose of the analgesic agent, buprenorphine. The operative region on the dorsal back was prepared by removing fur with clippers. Mouse skin is wiped in a single swipe motion 3X with an alcohol and two applications of 10% betadine. Mouse is draped then draped with a sterile cloth. Wounds are created by marking a rectangular box of defined size (1.5cm^2^) and then by cutting a full-thickness section along marked lines with sterile scissors. The wound is left open to allow for natural wound healing to occur. Pictures are taken every 2-3 days to monitor for wound healing (scabs are removed by treating animals with Vaseline 1 day prior to taking pictures to loosen up attachment). Wounds and scars were measured using Image J. Scale was set for every picture based on ruler placed next to animal. We did not see a noticeable difference in wound healing between male and female (*data not shown*) and ensured that all experiments contain both male and female animals age and sex matched to the best of our abilities. Following injection of tamoxifen, the mice did not display any signs of distress, and remained healthy without weight loss or other adverse phenotype.

#### Fibroblast isolation

Protocol modified from previously publication.^23^ Briefly, dorsal area of untreated mice was shaved and a small (1cm X 1cm) full thickness section was cut out and briefly dipped in 70% ethanol. After air drying the skin was cut into small pieces and place in a 2mL screw top tube with a Collagenase D/Pronase Enzyme solution at 37C with agitation (200rpm) for 90 mins. Digested samples are passed through a 70uM strainer and washed with complete medium. Cells are spun and resuspended in complete medium containing penicillin/streptomycin and amphotericin B. Replace the medium after 72 hours and subculture when culture reaches 70% confluency. Passaged regularly.

#### Fibroblast transfer experiments

Cultured primary cells were harvested with trypsin (0.25%) prior to the 3^rd^ passage, washed 2 times with sterile PBS and counted. Cells were resuspended in sterile PBS to a concentration of 5X10^4^/uL for a total of 5X10^6^ per wound injected intradermally into the 4 sides of the wound bed (∼1.25X10^6^ cells per side). The number of fibroblasts injected into the wound bed was determined according to a previously published manuscript.^24^ Zhang et al. showed improved wound healing with 4x10^5^ fibroblast cells implanted into 6 mm wounds (∼0.28 cm^2^). Our wound size (1.5x1.5cm) targeted an area of ∼2.25cm^2^, which would require ∼4 x 10^6^ cells. We chose to give 20% more cells since it was difficult to ensure all cells would remain in the wounds. Pictures were taken every few days to calculate the effects on wound healing. PBS alone was used as a control in mice not receiving fibroblasts. We also included experiments comparing Col5a1 CKO fibroblasts into WT and Col5a1 CKO as a controls and did not observe any improvement in WT or Col5a1 CKO wound healing (Figure S2).

#### Treatment of animals with Cilengitide

Cilengitide was purchased from (MCE, #HY-16141). Mice were given an intraperitoneal injection of Cilengitide (20mg/kg) diluted in PBS intraperitoneally daily starting 24 hours prior to wounding until day of harvest. This dose was selected because we previously found that it improved lesion size following cardiac injury.^11^ Pictures were taken every few days to calculate the effects on wound healing. PBS was used as a vehicle control. This experiment.

### Method details

#### Wound size calculation

Wounds sizes were calculated using Image J. Each wound picture contained a ruler for standardization and the scale was set for each picture before measurement was obtained.

#### Antibodies

The following primary antibodies were used for immunostaining: rabbit anti-CD45 (1:100, Abcam, ab10558); rabbit anti-Cd11b (1:100, Abcam, ab133357); rabbit anti-CD68 I (1:100, Abcam, ab125212), aVb3 (1:100, R&D MAB3050), aVb5 (1:100, Bioss, bs-1356R). Secondary Donkey anti-rabbit IgG (H+L) AlexaFluor™ Plus 488 (1:250, Invitrogen A32790) and .

#### Bulk RNA-seq

For bulk RNA-seq, a 6mm punch biopsy was taken from the middle of wound or scar cut into small pieces with sterile scissors and placed in RLT lysis buffer. Chrome beads were added and tissue was homogenized using the TissueLyser LT (Qiagen). Total RNA was prepared using RNeasy kit (Qiagen). Strand-specific libraries were generated using 400 ng of total RNA using the TruSeq stranded RNA Sample Preparation Kit (Illumina). cDNA libraries were single-end sequenced (50bp) on Illumina HiSeq 4000.

#### RNA-seq analysis

Reads were aligned to the mouse genome (NCBI37/mm9 build) with Hisat2 by allowing reads to be aligned once with up to two mismatches per read. SeqMonk (Babraham Bioinformatics) was used to quantify against the exons of Refseq genes and RPKM values for genes were calculated as described.^25^ Briefly, RPKM = (“Number of reads mapped to exons of a gene”/ “Exon length in kilo-base x Total mapped reads in million”). Significantly different gene lists were determined using the Bioconductor DESeq-2 package in R. A gene was considered significant if it met all of the following criteria: The maximum average RPKM must reach 1 in any group, the fold induction level reached 3-fold, and the induced expression level was consistently different (*P*<0.05). A k-means cluster (K=4) with Euclidean distance measurement and 100 iterations was performed with mean centered log2 RPKM of the list of significantly induced genes in CKO wound compared to WT wound on day 19 using Cluster 3.0.^21^ Initial 4 clusters were reduced 2 three by manual curation of the clusters. Two clusters which were enriched for “Epidermis regeneration genes” and “Inflammatory response genes” are represented in the figures. All heat maps displayed are of averaged RPKMs from at least three independent biological replicates that were log2 normalized and centered around mean in Cluster 3.0. Heat maps were generated using Microsoft Excel using a 3-color formatting rule based on percentiles (10, 50, 90). Z-scores were calculated by taking the average score for a list of genes by taking the raw RPKM value, subtracting the mean, and dividing by the standard deviation for each gene in the list. Gene sets from each cluster were used to find enrichment for Gene Ontology (GO) terms for biological processes using Enrichr software.^19^ Inflammatory genes enrichment in the clusters B was verified by comparing TLR4 induced RNA-seq data sets generated in primary macrophages.^25^

#### Total collagen content

Collagen Content was measure using Sircol Insoluble Collagen Assay (Biocolor S2000). A small piece of wound (∼30mg) was homogenized in a 0.1mg/mL pepsin/ 0.5M acetic acid solution and placed at 4C for 24hrs to extract soluble collagen. Lysates were spun down at 12,000 rpm for 10 minutes, supernatants were removed and placed in another tube for analysis using the same kit. The Sircol procedure was then used to extract insoluble collagen. Tissue residues were incubated with Fragmentation Reagent at 65C for 3 hours with vortex every 30 minutes. Samples were centrifuged for 12,000 rpm for 10 min. Supernatants from both the insoluble and soluble samples were diluted 1:1 with water and then mixed with 1mL of Sircol Dye Reagent for 30 minutes at room temperature with gentile shaking every 10 min. Samples were centrifuged for 10 minutes at 12K rpm, supernatants were discarded and precipitates were washed with 750uL of ice-cold Acid salt Wash reagent. Samples were again centrifuged at 12,000 rpm for 10 min and pellet was resuspended in 500uL of Alkali Reagent. 200 ul of collagen solution was transferred to a 96 well place along with 200uL of collagen standards and absorbance was measured at 550nm using Synergy H1 microplate reader (BioTek) within 1 hour of completing the collagen isolation. Concentrations were determined based on the standard curve and total collagen content was determined by adding the insoluble collagen value with the soluble collagen value.

#### Histological studies

Skin was harvested from euthanized animals and fixed in 4% formalin for 24 hours, subsequently tissues were washed in PBS, placed in 70% ethanol and brought to the Translational Pathology Core Laboratory (TPCL) core at UCLA for paraffin embedding. Samples were sectioned with 5-8mm-thickness for subsequent staining. Sections were blocked in 10% species-specific normal serum or Isotype control (Rabbit IgG (monoclonal for CD11b and polyclonal for CD45) in 1%BSA/PCS for 2 hours and primary antibodies diluted in 1%BSA/PBS at 4C overnight. Secondary antibodies were diluted in 1%BSA/PBS and incubated with sections for at least 1 hour. Slides were mounted with VECTASHIELD PLUS antifade mounting medium with DAPI (Vector Laboratories, H-2000-10). Images were taken using Nikon Eclipse Ti2 confocal microscopy (Nikon USA). Isotype control images can be seen in Figure S3.

For Masson Trichrome staining, sections were stained using Masson Trichrome Stain kit (Thermo Scientific, 87019). Briefly, slides were de-paraffinized and placed in Bouins Fluid overnight at room temperature. Slides were then stained sequentially with working Weigert’s Hematoxylin, Biebrich Scarlet-Acid Fuchsin, Phsphomolybdi/Phosphotugstic Acid, Aniline Blue Stain, 1% Acetic Acid, 3 changes of Absolute Alcohol, Xylene and mounted with Permount Mounting Medium (Fisher Scientific, SP15100).

#### Transmission electron microscopy

Samples were visualized according to.^11^ Briefly, samples were fixed in 2% glutaraldehyde in PBS at 4C for 3 hours and embedded in low-viscosity resin. Plastic-embedded samples were sectioned using UCT ultramicrotome (Leica, Austria) and diamond knife (Diatome, Austria). Sections were cut 50-55nm thick and were mounted on home-made EM grid(s) with plastic-carbon support film, stained with saturated uranyl-acetate and Sato’s lead-citrate. Sections were imaged using JEM1200EX transmission electron microscope (JEOL, Japan) at 80 kV equipped with BioScan600W digital camera (Gatan, USA). Images were prepared for publication using Photoshop (Adobe, USA).

#### Flow cytometry

Wounded skin was digested using liberase for 30 minutes at 37C. Liberase was inactivated with RPMI with 10% FCS and digested tissue was passed through a 70uM strainer. Samples were washed 2X and counted. Cells were treated with Fc block for 10 minutes at room temperature followed by staining with aVb3 antibody for 10 minutes at room temperature. Cells were washed with PBS, centrifuged at 1180 rpm for 5 minutes. Cells were then incubated with diluted Goat Anit-Rabbit IgG H&L AlexaFluor 594 secondary antibody (1:200, Abcam, ab150080) for 10 minutes at room temperature. Cells were again washed with PBS, centrifuged at 1180 rpm for 5 minutes and fixed with 2% PFA. Cells were run on the Fortessa (BD Bioscience) and data was analyzed using Flowjo software.

#### Cytometric Bead Array (CBA)

BD^tm^ Cytometric Bead Array was used according to the manufacturer’s protocol (BD Biosciences, #560232). Briefly, 20mg of wounded tissue was homogenized in PE Buffer, spun down at 12000rpm for 10minutes and supernatants were frozen until time of assay. On the day of assay 50ul of homogenized skin sample supernatant was used for assay. The recommended amount of detection reagent and capture beads were used for samples and standards. Beads were run on the Fortessa (BD Bioscience) and data was analyzed using Flowjo software, amount of IL1β was calculated using a standard curve.

#### Atomic force microscopy

For atomic force microscopy (AFM) experiments were conducted similar to (Yokota et al Cell 2020). Briefly, wounded skin was dissected and mounted in OCT (Tissue-Tek, Sakura Finetek Torrance, CA, USA) and flash frozen in liquid nitrogen-cooled isopentane. Skin tissue cryosection (30mm) were mounted onto microscope slides with an adhesive coating (#SUMGP14 Matsunami Glass Ind. Ltd, Kishiwada Osaka, Japan). AFM measurements were performed on each section in PBS using a JPK Nanowizard 4A BioAFM with a 200x200x200 mmHybridStage (Bruker/JPK BioAFM Billerica, MA,USA) coupled to a Leica M205 steroscope (Leica Microsystems, Wetzlar, Germany). Skin sections were probed with AppNano SHOCONGG-TL cantilevers with a 10 mm silicon dioxide sphere (nominal freq (kHz) – 21(8-38), k(N/m) =0.14 (0.01-0.06); AppNano Mountain View, CA USA). To evaluate tissue stiffness, Young’s modulus was calculated using >100 AFM force curves and the Hertz-Sneddon model (Sneddon, 1965). Young’s modulus data were plotted and statistics calculated in GraphPad (Prism) software using Mann-Whitney test; since values did not pass normality test.

### Quantification and statistical analysis

All data is presented as mean and standard error. The n for each experiment is mentioned in the figure legends and always represents separate biological replicates. Statistical analysis was performed using GraphPad (Prism) software. Specific tests are mentioned in figure legends and were determined based on Shapiro-Wilk normality test.
