# Supplementary material for: Modulating the extracellular matrix to treat wound healing defects in Ehlers-Danlos syndrome
Source: iScience. 2024 Aug 6;27(9):110676. doi: 10.1016/j.isci.2024.110676 (PMC11389543; doi:10.1016/j.isci.2024.110676)
Supplement: Document S1. Figures S1–S4 [file mmc1.pdf]

iScience, Volume *27*

Supplemental information

Modulating the extracellular matrix to treat wound healing defects in Ehlers-Danlos syndrome

Kindra M. Kelly-Scumpia, Maani M. Archang, Prabhat K. Purbey, Tomohiro Yokota, Rimao Wu, Jackie McCourt, Shen Li, Rachelle H. Crosbie, Philip O. Scumpia, and Arjun Deb

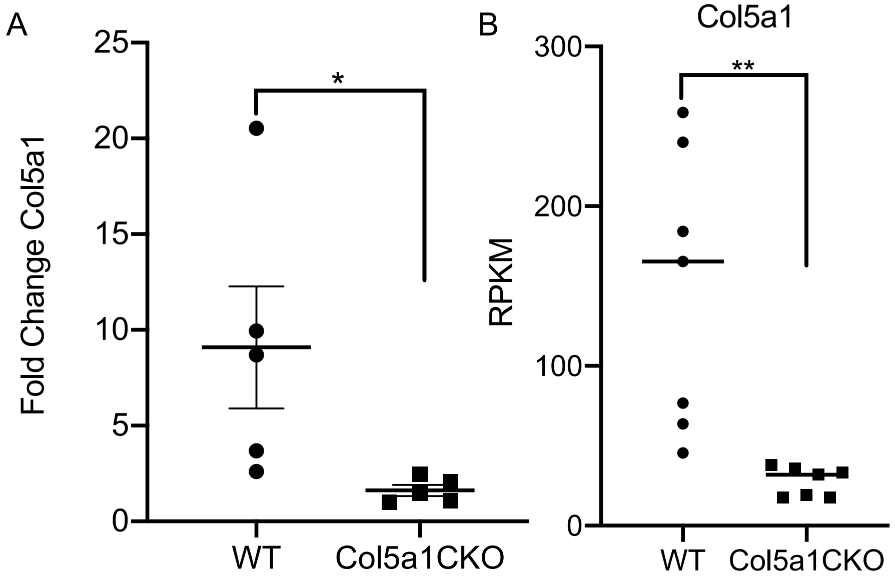

Supplemental Figure 1. Col5a1 is decreased in Col5a1CKO animals following tamoxifen treatment, related to Figure 1. A. PCR of WT and Col5a1CKO skin (Day 14) for Col5a1 showing a significant decrease in Col5a1 expression in Col5a1CKO animals (n=5, p<0.05 mann-whitney t-test). B. RPKM from bulk RNAseq at day 14 showing significant decrease in RPKM of Col5a1CKO skin (WT+vehicle n=7 (4M, 3F), Col5a1CKO+vehicle n=7 (3M, 4F), p<0.01 mann-whitney t-test), C. Heat map of most of the collagen genes showing decrease in Col5a1CKO animals compared to WT (Avg RPKM of mice in B).

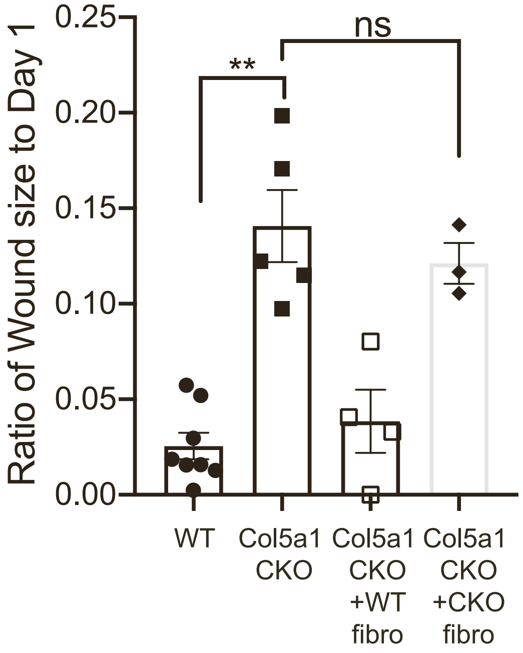

Supplemental Figure 2. Transfer of Col5a1CKO fibroblasts has no effect on wound healing in Col5a1CKO animals, related to Figure 3. Control experiments for the addition of WT fibroblasts. Col5a1CKO fibroblasts were cultured like WT fibroblasts and were transferred to the wound beds of Col5a1CKO animals as in Figure 3F. A total of 5X106 million fibroblasts were added, this is the same amount of WT fibroblasts added and saw a significant improvement in wound healing. We did not see improvement in wound healing in Col5a1CKO animals that received Col5a1CKO fibroblasts (n=8 WT, n=5 Col5a1CKO, n=4 Col5a1CKO+WT fibroblasts, n=3 Col5a1CKO+Col5a1CKO fibroblasts; ** $p<0.001$ Dunn's multiple comparison test)

isotype control for 2F (CD45)
Col5a1CKO IgG iso with-Goat anti rabbit_alexa488 secondary

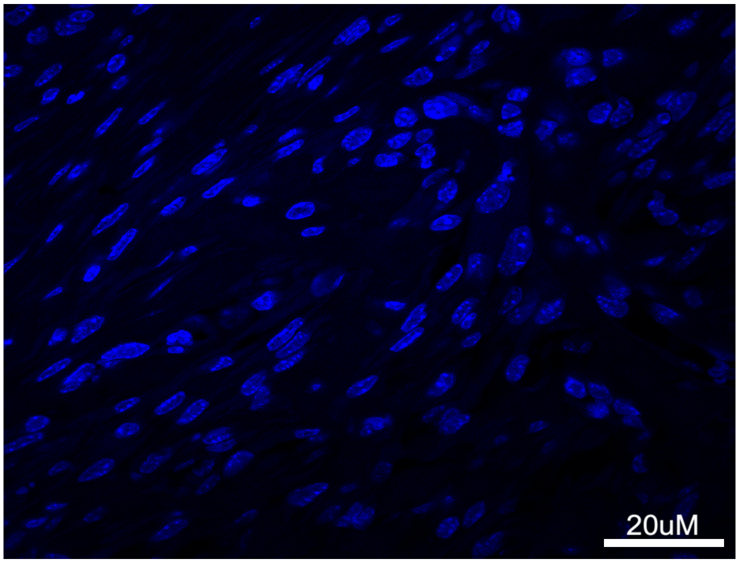

isotype control for 2F and4E (CD11b)
Col5a1CKO IgG iso with-Goat anti rabbit_alexa488 secondary

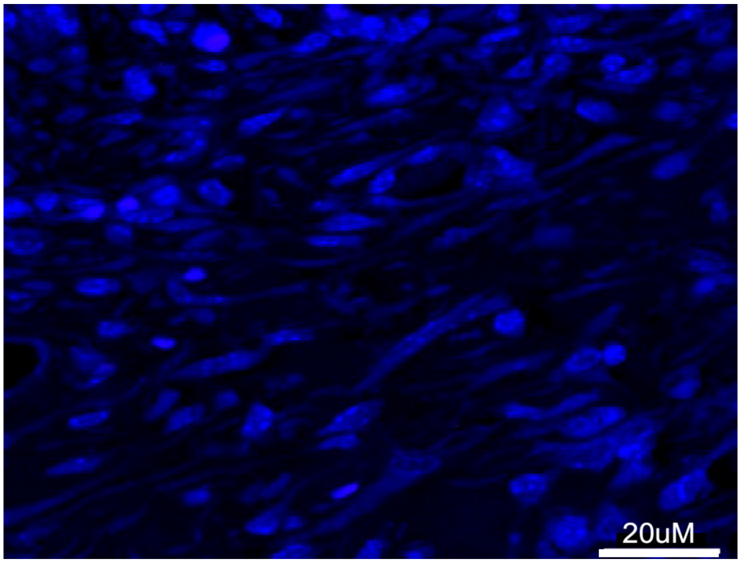

Supplemental Figure 3. Isotype controls for Immunofluorescence staining, related to Figure 2 and 4. A. Representative picture of isotype control staining for Figure 2F (of at least and n=3 per group). Voltages were set on isotype controls and no difference was seen between samples. B. Representative picture of isotype control staining for Figure 4E (of at least and n=3 per group). Voltages were set on isotype controls and no difference was seen between samples.

A

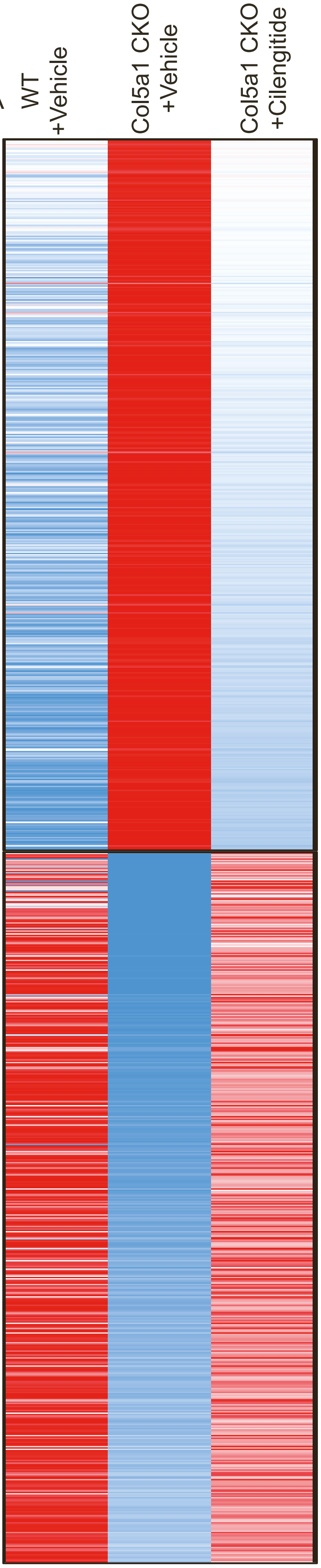

B

GO Biological Processes	Adjusted p-value	Genes
Positive Regulation Of Cytokine Production (GO:0001819)	1.45E-15	Cd86, Cd80, Hilpda, Tnf, Pik3cg, Tbk1, Dhx58, Flot1, Pde4b, Il12a, Ikbke, Il10, Fcer1g, Syk, Il15, Slc11a1, Il16, Cyba, Mmp8, Il17ra, Tlr1, Fgr, Il1a, Tyroby, Il23a, Ddit2, Il1b, Rara, Irf7, Irf5, Tlr6, Lcp1, Ltb, Tlr4, Tlr2, Cd274, Cebpb, Ebi3, Ptgs2, Agr, Malt1, C3, Clec7a, Hmox1, Nlrp3, Slamf6, Cd14, Egr1, Nos2, Osm, Isg15, Sorl1, Ccdc88b, Ptprc, Il17f, Cd244, Crlf2
Inflammatory Response (GO:0006954)	1.77E-15	Cebpb, Ptger2, C5ar1, Itgb2, Fpr1, Cxcr4, Cxcl1, Fpr2; Mefv, Cxcl3, Itgal, Tnf, Ager, Cxcl2, Thbs1, Pik3cg, Tbk1, Irak2, Adora3, Cxcr2, Ccrl2, Ccl4, C3ar1, Ccl3, Prok2, Olr1, Nlrp3, Cd14, Ccr5, Ccr1, Nos2, Slc11a1, Cyba, Ppbp, Fos, Nfkb2, Tlr1, Il1a, Il1b, Mapkapk2, Rel, Adam8, Tlr6, Fcgr2b, Tlr4, Cd44, Tlr2, Pf4
Regulation Of Tumor Necrosis Factor Production (GO:0032680)	1.52E-13	Cd84, Tnfaip3, Ptprj, Ager, Thbs1, Zfp36, Clec7a, Ccl3, Cd14, Cd33, Il10, Arg2, Syk, Nfkbil1, Cyba, Irak3, Mmp8, Lilrb4, Tlr1, Il1a, Tyrobp, Ptprc, Il23a, Oas2, Oas3, Mapkapk2, Rara, Ptpn6, Cd47, Lcp1, Tlr4, Pf4
Cytokine-Mediated Signaling Pathway (GO:0019221)	1.68E-13	Csf3, Csf3r, Ebi3, Ptprj, Csf2rb, Cxcl1, Il1rap, Cxcl3, Tnf, Csf2r1, Cxcl2, Il18rap, Irak2, Cxcr2, Ccl4, Stat4, Ccl3, Il12a, Cd300ff, Ccr1, Lyn, Egr1, Fcer1g, Syk Gpr35, Il15, Osm, Irak3, Ppbp, Traf1, Tnfrsf1b, Lilrb4, Il17ra, Nfkbia, Hck, Il1a, Ceacam1, Aim2, Oas2, Il1b, Il17f, Irf5, Ptpn6, Il7r, Crlf2, Pf4, Birc3
Regulation Of Interleukin-6 Production (GO:0032675)	1.16E-11	Cd84, Tnfaip3, Tnf, Agr, Clec71, Nckap1l, Il10, Syk, Nos, Foxj1, Cyba, Il16, Irak3, Mmp8, Lilrb4, Il17ra, Tlr1, Il1a, Nlrp12, Tyroby, Il1b, Mapkapk2, Il17f, Ptpn6, Cd47, Lcp1, Tlr6, Tlr4, Tlr2,

C

GO Biological Processes	Adjusted p-value	Genes
Epidermis Development (GO:0008544)	1.71E-05	Dsp, Col17a1, Sprr3, Wnt10a, Crapb2, Krt2, Klk14, Grhl3, Krt5, Ovol1, Evpl, Krt85, Klk7, Ovol2, Spint 5, Fabp5, Krt 14, Tgm5
Sterol Biosynthetic Process (GO:0016126)	9.63E-05	Sqle, Nsdhl, Mvk, Pmvk, Dhcr24, Mvd, Hsd17b7, Lss, Tm7sf2, Sdft1
Lipid Biosynthetic Process (GO: 0008610)	1.06E-04	Acss2, Mvk, Prkaa2, Evovl4, Acsl1, Srd5a1, Mboat1, Lss, Acacb, Acaca, Agpat3, Dolpp1, Fasn, Mvd, Fdft1
Secondary Alcohol Biosynthetic Process (GO:1902653)	1.34E-04	Nsdhl, Mvk, Pmvk, Dhcr24, Mvd, Hsd17b7, Lss, Tm7sf2, Fdft1
Cholesterol Biosynthetic Process (GOL0006695)	1.61E-04	Nsdhl, Mvk, Pmvk, Dhcr24, Mvd, Hsd17b7, Lss, Tm7sf2, Fdft1

Supplemental Figure 4. Bulk RNAseq of WT+vehicle, Col5a1CKO+vehicle and Col5a1CKO+cilengitide (n=4 per group), related to Figure 3 and 4. Gene expression of Col5a1CKO+vehicle was compared with Col5a1CKO+cilengitid to obtain Deseq values and adjusted P-values using R studio. Based on an adjusted p-value of <0.05 and a FC of >=2 we obtained a gene list of 3001 genes that met this criteria. Data was normalized using Cluster and a k-means cluster was performed to obtain 4 different clusters. A. Cluster 1 and 2 displayed the differences between Col5a1CKO+vehicle vs Col5a1CKO+cilengitide as seen in the heat map B. Enrichr analysis of Cluster 1 shows a consistent inflammatory signature in Col5a1CKO animals compared to WT animals and WT and Col5aCKO+cilengitide show a similar response. C. Enrichr analysis of Cluster 2 shows a decrease in expression of epidermis development genes, including many Keratin genes, and this pathway in increased in Col5a1CKO+cilengitide animals similar to WT+vehicle animals.
